# Editorial: Methods and protocols in brain stimulation

**DOI:** 10.3389/fnhum.2023.1208260

**Published:** 2023-05-10

**Authors:** Sandra Carvalho, Fa-Hsuan Lin

**Affiliations:** ^1^Translational Neuropsychology Lab, Department of Education and Psychology, William James Center for Research (WJCR), University of Aveiro, Campus Universitário de Santiago, Aveiro, Portugal; ^2^Sunnybrook Research Institute, Toronto, ON, Canada; ^3^Department of Medical Biophysics, University of Toronto, Toronto, ON, Canada; ^4^Institute of Medical Science, University of Toronto, Toronto, ON, Canada

**Keywords:** non-invasive brain stimulation (NIBS), TMS, tDCS, tACS, protocols of brain stimulation

The use of non-invasive brain stimulation (NIBS) techniques to study the brain has increased significantly in recent decades. It has become one of the most accepted therapeutic approaches and powerful tools in treating neurological and psychiatric disorders. NIBS, such as Transcranial magnetic stimulation (TMS) and transcranial direct current stimulation (tDCS), have been proven effective in several clinical conditions, such as major depressive disorders, stroke, and to improve addition/craving and cognition, in both young and geriatric populations (Yavari et al., 2016; Lefaucheur et al., 2020; Fregni et al., 2021; Teixeira-Santos et al., 2022). However, methods and protocols of brain stimulation are very heterogeneous and further research is needed to fine tune the modulatory effects of NIBS in the brain.

In this research topic of methods and protocols of brain stimulation, five original research articles address various protocols of NIBS, such a study protocol for geriatric depression, a perspective article on how to test the association between baseline performance and effects of NIBS, and clinical trials discussing methods of brain stimulation on stroke and sleep quality.

Although the use of rTMS for the treatment of depression in adults is relatively well established, there is still the need to develop and test new ways of rTMS in geriatric depression. Theta-burst stimulation (TBS) is a form of rTMS that mimics the rhythms of brain activity and uses short bursts of high-frequency stimulation, with bursts of stimulation about five times per second (Blumberger et al., 2018). The main advantage of TBS over conventional rTMS is that sessions of TBS are short (3–12 min TBS vs. 20–30 min rTMS) and potentially with non-inferior clinical results (Blumberger et al., 2018). Thus, Valiengo et al. propose an innovative parallel, randomized, sham-controlled trial to study the efficacy of active versus sham TBS in elderly subjects with geriatric depression, assessing both clinical and biomarkers (the brain-derived neurotrophic factor, BDNF). The results add to the current literature on the treatment of geriatric depression. They may result in short-term clinical gains in this population, especially for those who cannot tolerate antidepressants or are resistant to standard treatments. Furthermore, the study of the effects on specific biomarkers will improve the understanding of the neurobiological mechanisms of depression and their relationship to the treatment.

There is a substantial body of evidence showing that the behavioral impact of NIBS can dramatically vary by various factors, such as stimulation frequency, electrical density timing of stimulation, and inter-individual differences in baseline performance. Thus, it is crucial to evaluate how baseline levels, brain states, and brain state dependency can impact the behavioral outcomes of brain stimulation. To date, only a few studies consider the treatment effect on baseline performance. Most studies use correlation or categorization approaches to establish the relationship between output power and post-stimulus changes. The first one consists of regressing or correlating the magnitude of the stimulation effect (defined as effective TMS/tDCS condition performance minus baseline/sham condition performance) with baseline performance (sham stimulation) [see, for instance, Wu et al. (2021)]. In the second approach, participants are classified based on baseline performance or median split. Then, behavior outcomes after the stimulation(defined as active TMS/tDCS status minus baseline) are compared. Across two subgroups ('low' and 'high' performers) (Silvanto et al., 2018), Lega et al. discussed the associated bias possibly related to these two approaches. In this work, they showed how the baseline performance predicts the effects of NIBS. They further analyzed the relationship between baseline and NIBS effects. In addition, they showed that mathematical combinations and regression to the mean could have a significant bias on estimates, leading to highly skewed conclusions even when the null hypothesis is true.

The heterogeneity of NIBS results may not be only determined by the clinical profile and baseline performance but also due to protocol heterogeneity. Thereby, a study by Chen et al. examined both clinical improvement and alpha (α) rhythm changes in resting-state electroencephalogram (rs-EEG) induced by three tDCS protocols in patients with chronic ischemic stroke. In this single-blind randomized crossover design, a total of nineteen patients received four experimental sessions with four tDCS protocols: anodal tDCS (atDCS), cathodal tDCS (ctDCS), bilateral tDCS (bi-tDCS), and sham tDCS. Both clinical and rs-EEG were assessed before and after the tDCS session. The results showed that the three tDCS protocols differentially modulated α-EEG. AtDCS, especially the low-α rhythms (8–10 Hz), increased the α power at focal regions in the central and distal regions of the frontal and parietal lobes, especially the low α (8–10 Hz). Bi-tDCS modulated mainly high-α rhythms (10-13Hz). No modulatory effects on EEG rhythms were found after either ctDCS or sham. In addition, clinical factors of post-stroke time and the degree of motor impairment were found to be associated with a high-α change from atDCS and bi-tDCS. The modulatory effects persisted for up to 20 min without decay. This study suggested that different tDCS protocols have distinct modulatory effects on the brain, as assessed by α-rhythm EEG. The authors also suggested the dependency of a high-α on clinical features, such as post-stroke time and motor impairment.

In a within-subjects, randomized crossover design (with null-stimulation controlled) by Ayanampudi et al., two 15-min pre-sleep personalized transcranial alternating current stimulation (tACS) was applied in 25 volunteers to modulate sleep-dependent neuronal activity in the theta/alpha frequency band. Specifically, they had fixed tACS patterns at 5 and 10 Hz and personalized tACS approach with the stimulation frequencies determined by the individual's peak EEG frequencies in the 4–6 Hz and 9–11 Hz bands. Results showed that personalized tACS extended sleep duration by 22 min, in contrast to the 19 minutes in the fixed tACS protocol. Fixed stimulation, compared to control, did not significantly prolong the sleep duration. Concerning falling asleep, personalized tACS reduced time to onset by 28% compared with fixed tACS. Personalized tACS also improved sleep duration by 33 min for the group with poor sleep quality, compared to both control and fixed tACS. The results of this pilot study have important implications for the design of future clinical trials by showing the therapeutic effects of personalized tACS on sleep, including in individuals with insomnia.

Another way to test new brain stimulation protocols is to combine two NIBS that have been tested separately for their effects on a specific clinical condition. Thus, in the study by Quin and colleagues, they tested the effects of TMS combined with peripheral repetitive magnetic stimulation (rPMS) on limb spasticity in subjects with post-stroke spasticity (PSS) on clinical recovery assessed by clinical scales and by pre- and post-stimulus resting-state brain activity using resting-state functional magnetic resonance imaging. A total of forty-nine PSS subjects were randomly assigned to one of three study groups: (1) Combined (LF-rTMS and rPMS); (2) Low-frequency rTMS (LF-rTMS), and (3) Control (routine rehabilitation). Results showed that all participants had a decrease in the MAS scores and an increase in both FMA-UE and MBI scores. However, the combined group had significant improvement in motor function and relieved spasticity in PSS. In addition, the combined groups also showed the increased amplitude of low-frequency fluctuation (ALFF) values over the right supplementary motor area, right middle frontal gyrus, and right cerebellum, and reduced ALFF values over the right post-central gyrus. When comparing ALFF between the three groups, the results showed that the combined method, compared with LF rTMS and control, resulted in increased ALFF values in the right cerebellum and decreased values in the frontoparietal cortex. The results provide evidence of the clinical efficacy of combining LF rTMs with rPMS to improve spasticity state and motor function in subjects with PSS, possibly due to the activity regulation in the cerebellum and frontoparietal areas.

In sum, we summarized five original research articles addressing various protocols NIBS that investigated fundamental questions in brain stimulation research, such as the effects of personalized stimulation or the combination of different NIBS. More research is needed to facilitate the current NIBS protocols to achieve better results in both basic science and applications of brain stimulation.

## Author contributions

All authors listed have made a substantial, direct, and intellectual contribution to the work and approved it for publication.
